# Exploring the potential of the sit-to-stand test for self-assessment of physical condition in advanced knee osteoarthritis patients using computer vision

**DOI:** 10.3389/fpubh.2024.1348236

**Published:** 2024-02-07

**Authors:** Zhengkuan Zhao, Tao Yang, Chao Qin, Mingkuan Zhao, Fuhao Zhao, Bing Li, Jun Liu

**Affiliations:** ^1^Department of Joint, Tianjin Hospital, Tianjin, China; ^2^Tianjin Medical University, Tianjin, China; ^3^National Elite Institute of Engineering, Chongqing University, Chongqing, China; ^4^Department of Nephrology, Tianjin Hospital, Tianjin, China

**Keywords:** knee osteoarthritis, computer vision, stiffness, physical function, body pose, sit-to-stand, spatiotemporal information

## Abstract

**Introduction:**

Knee osteoarthritis (KOA) is a prevalent condition often associated with a decline in patients’ physical function. Objective self-assessment of physical conditions poses challenges for many advanced KOA patients. To address this, we explored the potential of a computer vision method to facilitate home-based physical function self-assessments.

**Methods:**

We developed and validated a simple at-home artificial intelligence approach to recognize joint stiffness levels and physical function in individuals with advanced KOA. One hundred and four knee osteoarthritis (KOA) patients were enrolled, and we employed the WOMAC score to evaluate their physical function and joint stiffness. Subsequently, patients independently recorded videos of five sit-to-stand tests in a home setting. Leveraging the AlphaPose and VideoPose algorithms, we extracted time-series data from these videos, capturing three-dimensional spatiotemporal information reflecting changes in key joint angles over time. To deepen our study, we conducted a quantitative analysis using the discrete wavelet transform (DWT), resulting in two wavelet coefficients: the approximation coefficients (cA) and the detail coefficients (cD).

**Results:**

Our analysis specifically focused on four crucial joint angles: “the right hip,” “right knee,” “left hip,” and “left knee.” Qualitative analysis revealed distinctions in the time-series data related to functional limitations and stiffness among patients with varying levels of KOA. In quantitative analysis, we observed variations in the cA among advanced KOA patients with different levels of physical function and joint stiffness. Furthermore, there were no significant differences in the cD between advanced KOA patients, demonstrating different levels of physical function and joint stiffness. It suggests that the primary difference in overall movement patterns lies in the varying degrees of joint stiffness and physical function among advanced KOA patients.

**Discussion:**

Our method, designed to be low-cost and user-friendly, effectively captures spatiotemporal information distinctions among advanced KOA patients with varying stiffness levels and functional limitations utilizing smartphones. This study provides compelling evidence for the potential of our approach in enabling self-assessment of physical condition in individuals with advanced knee osteoarthritis.

## Introduction

Knee osteoarthritis (KOA) represents a substantial global health concern, affecting over 500 million individuals worldwide and resulting in personal and societal implications (1). Advanced KOA patients frequently contend with varying degrees of diminished functionality (2–4). However, they often face challenges in objectively assessing their condition (5, 6). Many patients have difficulty assessing the extent of their physical condition decline, which often leads to a tendency to delay seeking medical attention or not actively pursuing treatment until their symptoms have reached an exceedingly severe stage. Thus, there is an urgent need to develop novel self-assessment methods for a straightforward and objective initial evaluation of patients’ physical condition in a home-based context. Through this initial assessment, patients can objectively determine the extent of their disease symptoms, thereby assisting them to make reasonable decisions and seek timely medical guidance.

The prevailing method for assessing KOA patients’ symptoms and functional capabilities involves utilizing rating scales, with the well-recognized The Western Ontario and McMaster Universities Osteoarthritis (WOMAC) scale being a prime example (7). This scale evaluates stiffness and physical joint function, and its effectiveness has been extensively validated (8–10), making it a standard clinical practice component. However, it is essential to acknowledge that these assessments typically require specialized guidance and a substantial time investment. Patients often face challenges in accurately and independently completing these assessments without proper guidance. This obstacle significantly hampers the potential for widespread adoption of remote self-assessments among individuals with knee osteoarthritis.

As knee osteoarthritis progresses, patients undergo a gradual decline in physical condition (11, 12). Previous research emphasizes the significance of physical function analysis as an objective measure for assessing the physical condition of KOA patients (13). Studies have shown that the five-times sit-to-stand (STS) test (14–16) can evaluate critical factors such as the external knee adduction moment and knee alignment in the frontal plane, contributing to assessing physical function in KOA patients (17–20). Moreover, the STS test’s straightforward and user-friendly nature allows for a rapid and easy assessment of subjects’ physical function (21, 22). Building on this foundation, we aimed to correlate the STS test with physical function presentation in advanced KOA patients. If proven valid, this attempt could establish correlations between objective measures of functional capacity and patients’ subjective perceptions.

Previous computer vision technology in medicine usually focused on the segmentation and recognition of medical images (23, 24). With advancements in computer vision technology, clinical motion analysis has overcome the constraints of traditional laboratory settings. Artificial intelligence techniques now enable the cost-effective and user-friendly extraction of essential features and information from movements (25, 26). This groundbreaking progress has paved the way for the widespread adoption of large-scale clinical motion analysis. In particular, the AlphaPose technique has emerged as an exceptionally efficient method for meticulously capturing crucial two-dimensional body pose details from camera footage (27). It demonstrated remarkable capabilities in locating key positions of the whole body, even in the presence of inaccurate bounding boxes and redundant detections. Compared to the OpenPose method widely used in previous research, AlphaPose achieves higher accuracy (28, 29). Furthermore, The VideoPose approach, an open-source video-based three-dimensional pose estimation method, simplifies the complexities of learning from two-dimensional data. This method enables the acquisition of three-dimensional key body positions without requiring intricate feature learning processes, thanks to its foundation on time-dilated convolutional networks, which exhibit strong performance in dynamic environments (30–32). The integration of these methods empowers the extraction and comprehensive analysis of spatiotemporal motion insights derived from camera-generated data. These significantly reduce costs and lower the technical barriers traditionally associated with motion analysis.

Our study marks the initial attempt to utilize a smartphone video-based computer vision technique to discriminate differences in spatiotemporal information among advanced knee osteoarthritis (KOA) patients exhibiting varying degrees of stiffness and physical function. Our primary objective is to explore the correlation between these objective clinical data and patients’ subjective perceptions, including stiffness and physical functionality, thereby evaluating the potential of utilizing physical function test videos as an automated tool for patients’ self-assessments. If proven feasible, This research serves not only as an endeavor to standardize the self-assessment criteria for patients but also introduces a feasible idea to simplify and facilitate the remote self-assessment of advanced KOA patients.

## Methods

### Patients selection

Our study, approved by the Institutional Review Board (IRB 2023 Medical Ethics Review 066), obtained written informed consent from all participants. One hundred and fourteen subjects were recruited from Tianjin Hospital between May and July 2023. Inclusion criteria required advanced KOA patients with at least one knee graded three or higher on the Kellgren-Lawrence (KL) scale. Exclusion criteria encompassed: (1) history of lower extremity joint or back injury or surgery; (2) presence of hip, spine, or ankle diseases, including osteoarthritis; (3) lower extremity trauma or intra-articular treatment within 3 months before study enrollment; (4) frequent reliance on adaptive walking aids; and (5) presence of depression, cognitive impairment, or other neurological disorders affecting physical function. Each participant underwent a physical function assessment and a comprehensive knee radiograph of the entire limb. Two experts independently assessed all radiographs using the KL grading scale to evaluate KOA severity. After applying exclusion criteria, the study included 104 participants.

### Data collection

**Figure 1:**
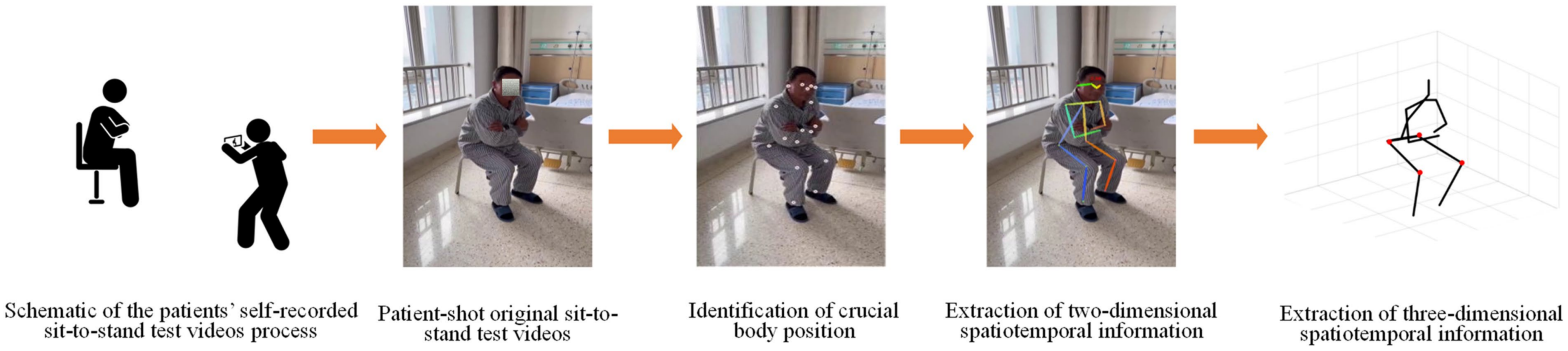
Schematic process of extracting sit-to-stand (STS) test data. Participants followed instructions to complete the STS test, recording full-body movements in videos. Subsequently, we used AlphaPose to extract crucial body points and generate two-dimensional positions from the videos. Finally, VideoPose was employed to estimate three-dimensional body positions based on the extracted two-dimensional pose information.

After obtaining informed consent, participants were provided instructional videos and written guidelines for the five-time STS test. This assessment involved participants crossing their arms in front of their chests and performing five consecutive sit-to-stand movements as rapidly as possible (15). Participants were instructed to record and submit corresponding videos independently. There were no recording angles or distance restrictions, aiming to maximize clinical applicability. The only requirement was to capture all their body movements during the shot comprehensively. Following the recording, participants reviewed their videos, and these materials were then transmitted to the research team. Subsequently, a subsequent manual review of the videos was conducted, excluding subjects who did not record the entire test process or who did not record the entire test body position. In the end, we obtained data from 104 subjects. For a visual representation of the data collection and analysis process, please refer to Figure 1.

The participants underwent assessments of WOMAC (9). Additionally, we collected essential demographic data, including age, sex, height, and weight. The corresponding body mass index (BMI) was calculated using the provided height and weight information.

### The criteria for stiffness and physical function

The severity of stiffness and physical function was evaluated using WOMAC scale, a widely utilized tool for assessing knee osteoarthritis (33). Stiffness was categorized into two levels based on scores: mild stiffness (0 < score < 4) and severe stiffness (score ≥ 4). Physical function was classified into two levels based on scores: mild limitation (0 < score < 35) and severe limitation (score ≥ 35) (34).

### Methods of obtaining time series data

We employed AlphaPose, a lightweight single-person pose estimation model, to extract spatial information about body poses from the original video footage. AlphaPose boasts a superior combination of rapid processing speed and heightened accuracy. This model has demonstrated exceptional proficiency in identifying critical body landmarks, even under imprecise bounding boxes and surplus detections (27). Using AlphaPose, we obtained two-dimensional image plane coordinates for 17 pivotal positions within each video frame, facilitating comprehensive tracking of essential body poses. Further details on the schematic framework of AlphaPose can be found in the Supplementary Materials. To assess the efficacy of the AlphaPose method in extracting positions, we evaluated the quality of the merged image with the original image and the visualized body key position image in the dataset.

Subsequently, to obtain the three-dimensional characteristics from the two-dimensional information of the patients, we employed VideoPose, an open-source video-based three-dimensional pose estimation approach. VideoPose utilizes a temporal dilated convolutional model with a temporal field of perception spanning multiple frame(32)s. This approach effectively mitigates the learning complexity associated with the two-dimensional information model, facilitating the acquisition of three-dimensional information without the need for complicated feature learning processes. Further details about the schematic framework of VideoPose are available in the Supplementary Materials.

To enhance the clinical applicability of our study, we intentionally avoided imposing restrictions on the angles and positions of the captured videos. Consequently, variations in the length proportions of body segments may occur, potentially leading to a less meaningful interpretation of absolute coordinate values extracted from the videos. However, it is crucial to note that the relative positional relationships between real-world scenarios and the obtained absolute coordinates remain consistent across recordings. Consequently, the key pose angles maintain consistency regardless of the varied recording angles and positions.

Our focus centered on four key position angles crucial for reflecting the subjects’ physical function: “right hip,” “right knee,” “left hip,” and “left knee” (35–38). The “right hip” and “left hip” angles represent the angles between the respective hip joints, while the “right knee” and “left knee” angles depict the angles of the corresponding knee joints. Utilizing cosine trigonometry, we derived the extension-flexion angles of these joints over time. This approach allowed us to capture spatiotemporal information regarding angular changes within three-dimensional space. As a result, we could objectively evaluate participants’ physical function differences, thereby identifying disparities in physical condition among subjects during their STS tests.

### Processing of the time series data

The horizontal axis represents the sampling points, while the vertical axis represents the key position angles. To maintain consistency across the dataset, for each time series data exceeding a size of 400, we evenly removed a portion to bring it down to 400. It ensured that all data had a uniform format and length. The uniformity in data format and length facilitated the comparison of time series data differences among different populations in subsequent stages.

### Comparison of time series data

When conducting data analysis, considering the affected knee is crucial, as knees with osteoarthritis may exhibit different behavior than unaffected ones. Among the 104 patients, we collected data from 77 individuals with advanced left knee osteoarthritis and 84 with advanced right knee osteoarthritis. It is essential to take the affected side into account during analysis. Consequently, in the follow-up analysis, we selected time series data from patients with advanced left knee osteoarthritis for the “left knee” and “left hip” positions. Similarly, we select data from patients with advanced right knee osteoarthritis for the “right knee” and “right hip” positions.

We began our analysis by qualitatively examining the time series data from the four key locations in the datasets, visualizing the time series data for each subject. Simultaneously, we generated average change curves to assess the overall time series data differences between different patients during the tests.

We then used the pywt.dwt function to perform a discrete wavelet transform (DWT) on the curves (39, 40), resulting in two wavelet coefficients: the approximation coefficients (cA) and the detail coefficients (cD). cA captures low-frequency components, representing information related to long-term and gradual changes, while cD represents high-frequency components, encompassing details concerning short-term and rapid changes. This decomposition enables multiresolution analysis, facilitating a quantitative examination of disparities in the continuous change processes during the STS test.

We computed the absolute differences in both cA and cD at each site for the mean curves of various populations of KOA patients. This calculation provided a quantitative measure of dissimilarity between the mean curves. It is essential to note that, given the limited clinical significance of analyzing cA or cD values in isolation, our study emphasizes discussing trends in coefficient differences. Subsequently, we generated plots of the wavelet coefficients for each average curve, enabling direct visualization of the differences between mean curves in different groups.

All analyses were performed using Python 3.9 to ensure consistency in data processing and results for reproducibility.

## Results

### Participant characteristics

After review, 104 advanced KOA patients were enrolled in this study out of the initial 114 participants. The mean age of the study participants was 67.1 years, and the mean body mass index was 27.7 kg/m^2^. Among the 104 patients, data were collected from 77 patients with advanced left osteoarthritis and 84 patients with advanced right osteoarthritis. Table 1 summarizes the detailed demographic characteristics of the participants.

**Table 1:** Demographic characteristics of advanced knee osteoarthritis patients.

	Mild stiffness	Severe stiffness	Mild limitations of physical function	Severe limitations of physical function	Total
Subjects	*N* = 71	*N* = 33	*N* = 56	*N* = 48	*N* = 104
**Age (years)**					
Mean (SD)	67.6 (5.5)	67.2 (5.9)	67.5 (5.6)	67.5 (5.6)	67.1 (5.6)
Min, Max	53–81	53–78	53–81	53–79	53–81
**BMI (m/kg**^**2**^**)**					
Mean (SD)	27.4 (3.3)	27.4 (3.2)	27.2 (3.1)	27.6 (3.4)	27.7 (3.3)
Min, Max	20.9–35.8	23.1–33.8	20.9–34.5	22.5–35.8	20.9–35.8
**Sex**					
Male	25 (35.2%)	8 (24.2%)	24 (42.9%)	9 (18.8%)	33 (31.7%)
Female	46 (64.8%)	25 (75.8%)	32 (57.1%)	39 (81.2%)	71 (68.3%)

Data are n (%) or mean (SD). *Proportions calculated using the corresponding subgroup number in “Population.” This table shows the mild joint stiffness group, severe joint stiffness, mild physical function limitations, severe physical function, and total demographic characteristics of advanced knee osteoarthritis patients.

### Obtained STS data

**Figure 2:**
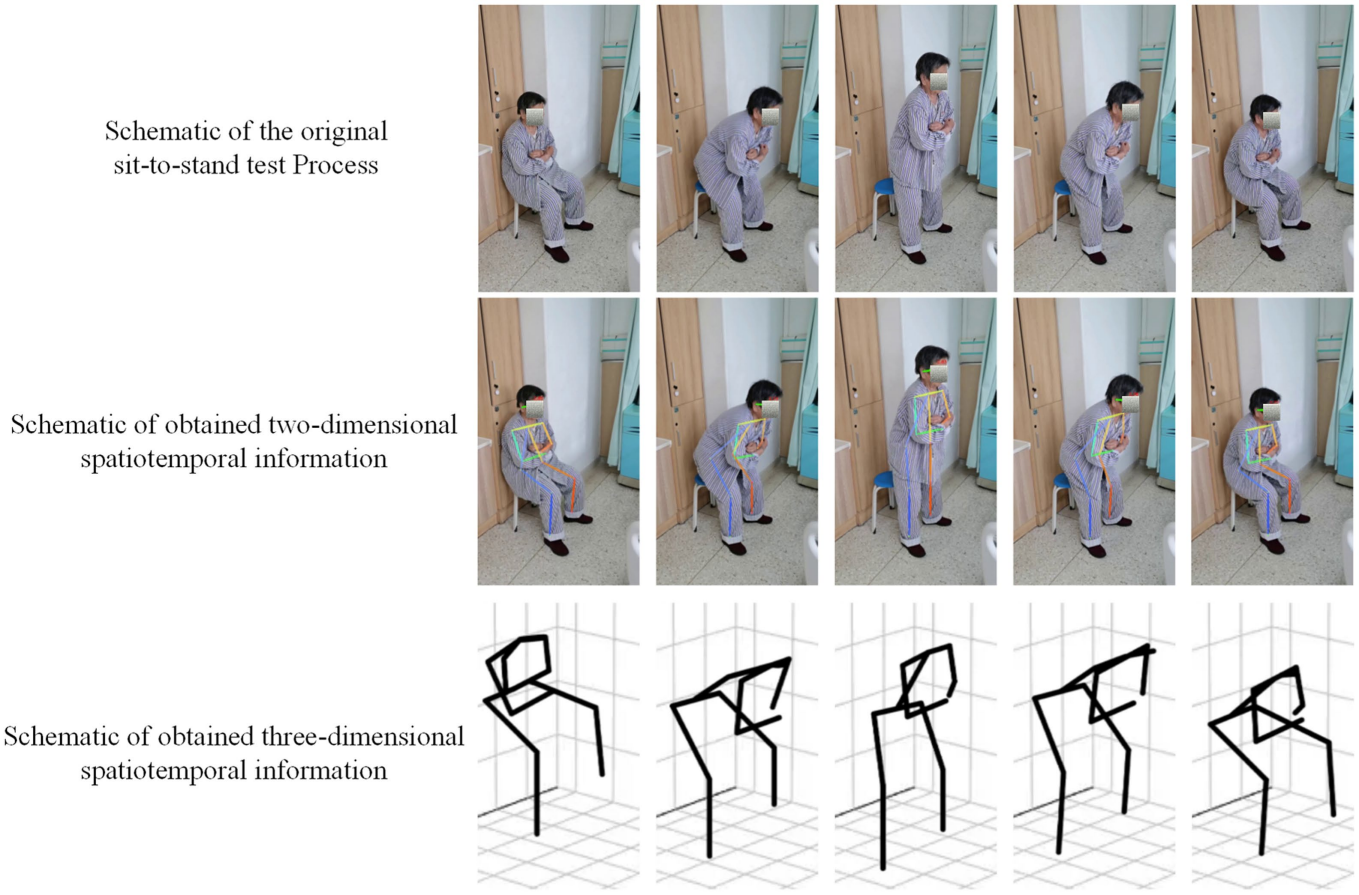
Schematic results of two-dimensional and three-dimensional body position extracted from the videos.

We extracted three-dimensional data and conducted manual reviews, revealing that combining AlphaPose and VideoPose is a practical approach for extracting spatial information from patients’ STS tests. Figure 2 provides a schematic representation of the result of extracting spatial information.

### Time series data qualitative analysis

The angular changes we extracted corresponded to the STS tests performed by the subjects. The results indicate that patients with lower stiffness levels and milder physical function limitations exhibited more pronounced angular changes in all key positions during the tests. Specifically, the angles of their “right hip,” “right knee,” “left hip,” and “left knee” showed a greater range of changes over time, with a more concentrated numerical distribution of angle changes across patients.

**Figure 3:**
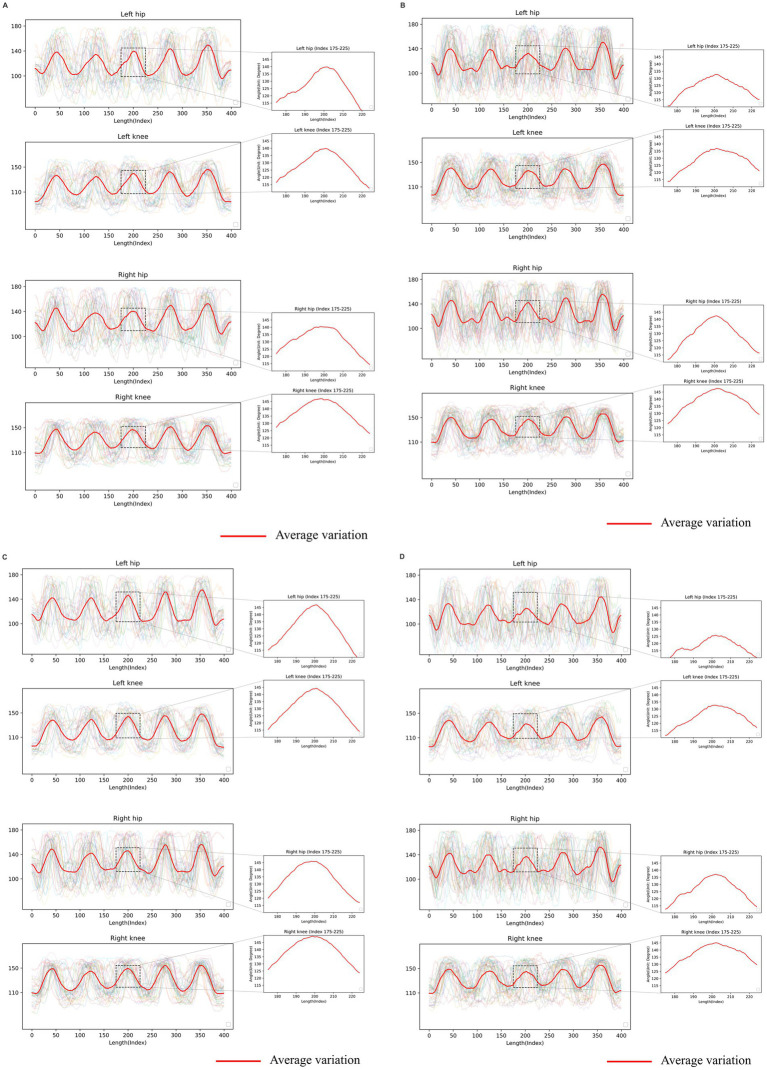
Visualized results from the sit-to-stand (STS) test. The graphical representation consists of a horizontal axis denoting the timeline and a vertical axis indicating the key position angle. The red line signifies the average variation observed in the dataset. The following provide time series data for different subsets of advanced knee osteoarthritis (KOA) patients: **(A)** Time series data of advanced knee osteoarthritis (KOA) patients with mild joint stiffness. **(B)** Time series data of advanced KOA patients with severe joint stiffness. **(C)** Time series data of advanced KOA patients with mild limitations of physical function. **(D)** Time series data of advanced KOA patients with severe limitations of physical function.

In contrast, patients with higher stiffness levels and more severe physical function limitations showed less angular change and a more dispersed distribution of angular change values. Notably, their angular changes were smaller and more unevenly distributed during the third sit-to-stand. The differences in angular change among patients with different physical functions throughout the test were significantly higher than those at the stiffness level. Moreover, more pronounced differences in variation regarding overall trends during movement were observed in hip joint angles compared to knee joints. Figure 3 illustrates the angular changes in key positions across participants, providing a visual comparison of the complete data and its average results.

### Time series data quantitative analysis

In the subsequent quantitative analysis, differences in the cA were observed between the two categories of patients, while the cD were very close between the two groups. Simultaneously, we found that the difference in the cA of the knee angle was more significant than that of the hip joint in patients with different stiffness levels. Conversely, the difference in the cA for the hip joint angle was more significant than that for the knee joint in patients with different physical functions.

**Figure 4:**
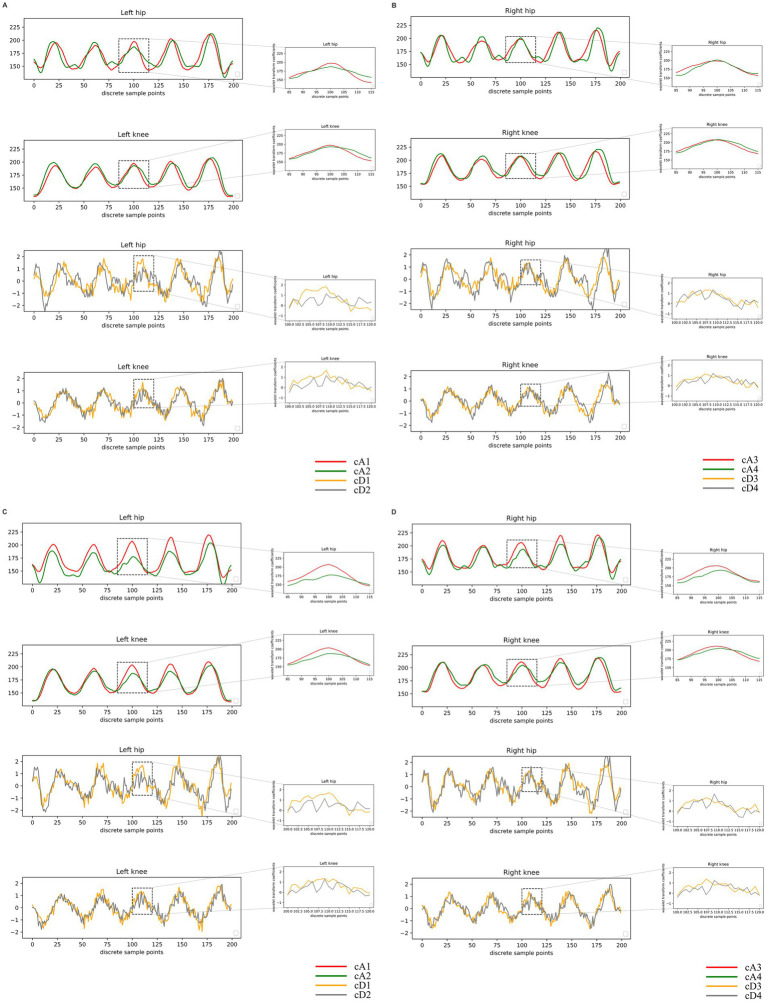
Visualized results of quantitative analysis. In the plots of the wavelet coefficients, the horizontal coordinates depict the indexes of the data points, signifying their positions on the averaged curves. The vertical coordinates represent the coefficient values derived from the discrete wavelet transform (DWT) process. These values denote the various band components resulting from the DWT decomposition. **(A,B)** Presents the results of a quantitative analysis conducted on patients categorized by joint stiffness. The abbreviations used are cA1: approximation coefficients of the angular variation curves in advanced knee osteoarthritis (KOA) patients with mild joint stiffness; cA2: approximation coefficients of the angular variation curves in advanced KOA patients with severe joint stiffness; cD1: detail coefficients in advanced KOA patients with mild joint stiffness; cD2: detail coefficients in advanced KOA patients with severe joint stiffness. **(C,D)** Presents the results of a quantitative analysis conducted on patients categorized by physical function limitations. The abbreviations used are cA3: approximation coefficients of the angular variation curves in advanced knee osteoarthritis (KOA) patients with mild limitations of physical function; cA4: approximation coefficients of the angular variation curves in advanced KOA patients with severe limitations of physical function; cD3: detail coefficients in advanced KOA patients with mild limitations of physical function; cD4: detail coefficients in advanced KOA patients with severe limitations of physical function.

Our results suggest variations in time-series data between patients with different stiffness levels and physical function. Specifically, the quantitative differences in knee angle changes were more significant for patients with different degrees of stiffness. In contrast, the quantitative differences in hip angle changes were more significant for patients with different physical functions. Refer to Figure 4 and Table 2 for detailed quantitative analysis results.

**Table 2:** Quantitative analysis of coefficient differences.

	Stiffness		Physical function	
	cA	cD	cA	cD
Left hip	48.7	0.3	2267.6	10.0
Right hip	424.1	24.9	822.7	21.9
Left knee	617.9	10.6	478.5	6.1
Left knee	617.9	10.6	306.3	22.4

This table stiffness shows the results of the quantitative analysis of stiffness between mildly stiff patients and severely stiff patients. Physical function shows the results of the quantitative analysis of stiffness between mild limitations of physical function patients and severe limitations of physical function. cA and cD represent the absolute values of differences at each joint position of the approximation coefficients and detail coefficients of Different stiffness levels and different motor functions of patients.

## Discussion

Our research represents a novel endeavor in applying computer vision recognition technology to medicine. It distinguishes itself from prior studies that primarily focused on recognizing and segmenting medical images (41–43). We designed a remote self-assessment computer vision method for advanced KOA caused by articular cartilage degeneration in older adults. Subsequently, we discussed the feasibility and generalizability of this approach in evaluating advanced KOA patients with physical function and two classes of stiffness levels. In this study, participants were requested to self-record videos of their STS tests, simulating a home testing environment. We analyzed these videos to extract spatiotemporal information about key body locations and further examined the data for patients with different physical functions and stiffness levels. The primary goal of our study is to explore the potential of computer vision in enabling the assessment of advanced KOA patients through self-remote assessments in digital health.

Strict adherence to prescribed protocols poses a notable challenge in the self-teleassessment of subjects for digital health applications (44), such as maintaining a consistent chair height during the STS test. This challenge can impede the progress of home-based telehealth assessments. Our study tackles this issue by transforming extracted two-dimensional spatiotemporal information into its three-dimensional counterparts. This innovative approach mitigates camera angle, distance, and height restrictions when analyzing patients’ self-recorded STS test video data, enhancing the feasibility and applicability of patients’ self-assessments.

By reviewing previous clinical literature and integrating clinical practice, we observed that issues related to motor function or limb stiffness of KOA patients often manifest prominently in the knee and hip joints. Consequently, our study selected “right hip,” “right knee,” “left hip,” and “left knee” as observable indicators. It is important to note that we selected the most critical metrics for measurement but not the exclusive ones. Unlike previous studies that often selectively extract critical information for analysis and overlook the temporal evolution of physical states (45–48), especially in physical function assessment, our study adopts a comprehensive approach. We established a connection between time and the patient’s movement process. It is crucial to highlight that our study extends beyond focusing on a single movement indicator, encompassing the entire movement process. The results suggest that computer vision techniques can effectively recognize differences among patients with varying degrees of physical function and joint stiffness during self-assessment.

In our qualitative analysis, we observed more pronounced differences in hip joint angle changes among advanced KOA patients, exhibiting varying levels of physical function and joint stiffness compared to the knees. This observation could be attributed to the hip joint’s more significant angular changes during the STS test, making it more likely to manifest observable differences. Previous studies have highlighted that individuals with knee osteoarthritis employ diverse postures and maneuvers to alleviate knee joint restrictions during the transition from sitting to standing. This compensatory measure might elevate loads and motion pressures on the hip joint, leading to significant differences in hip motion compared to individuals without knee osteoarthritis (49, 50).

Our finding underscores the importance of focusing more than just analyzing the constrained knee in computer vision assessments of KOA patient self-assessments. Considering the state of hip motion is crucial for effective self-assessment in knee osteoarthritis patients. This discovery aligns with the results of Bennell et al. (51) and Thomas et al. (52), who similarly emphasized the significance of evaluating the hip joint in KOA patients. Typically, knee osteoarthritis patients show a more functional decline over time (53). Interestingly, our results show that advanced knee osteoarthritis patients exhibited less angular change in critical positions and a more uneven distribution during the third sit-to-stand. It may be related to the patient’s mental activity, as the test requires the patient to complete it in the shortest possible time, and the patient may speed up execution during the last few instances of the test.

In the quantitative analysis, we observed variations in the cA among advanced KOA patients with different levels of physical function and joint stiffness. These differences suggest distinctions in the low-frequency components related to changes in joint flexion angles, indicating that the two groups diverged in slower, trend-like patterns of overall joint movement. This encompasses factors such as joint stability and the extent of joint flexion. Specifically, a more significant difference in cA for the hip joint compared to the knee joint was identified in patients with diverse physical functions. Notably, the difference in cA was more pronounced in the knee than in the hip for patients with different stiffness levels, a finding that appears somewhat inconsistent with the quantitative assessment results. This inconsistency may be attributed to the fact that although the knee is the primary site of stiffness manifestation in KOA patients, individuals with joint stiffness often face a higher level of limitation in physical function (54, 55). Consequently, they are more likely to exhibit more significant differences in the hip during qualitative analysis. This underscores the need to consider the interrelationships between symptoms when evaluating KOA patients in subsequent studies. Furthermore, it highlights that quantitative difference analysis is necessary for patient self-assessments rather than solely relying on qualitative analysis.

Moreover, in our study, there were no significant differences in the cD between advanced KOA patients who demonstrated different levels of physical function and joint stiffness. It suggests that the two groups are similar regarding the high-frequency component of the change in joint flexion angle, implying that differences in the details of specific movements, such as rapid joint movements, tremors, or shudders occurring over relatively short time scales, were insignificant. This lack of distinction may be attributed to the difficulty subjects encountered in accurately capturing the specific details of their movements when using video recordings independently. It emphasizes that a comprehensive and holistic perspective may hold greater importance than focusing on localized details regarding patients’ brief self-assessments.

While our study represents an innovative endeavor in the field, it is necessary to acknowledge its limitations. In qualitative analyses, our focus centered on discussing coefficient differences since the values of approximation or detail coefficients alone hold limited clinical significance. Although qualitative analysis indicates that computer vision techniques can recognize variations among patients with distinct physical function and stiffness levels, the precise magnitude of these differences remained undetermined. Future studies should explore and quantify the extent of these differences. Additionally, as the subjects in the study were all Chinese patients at the clinical center, the generalizability of our findings requires validation through data collected from multiple centers. It is important to note that while our study simplifies potential challenges in patient self-assessment video collection, differences may still exist between patient self-assessment acquisition data and data obtained in a professional setting or with specialized equipment. The implications of these differences must be considered when evaluating the feasibility of more complex applications of patient self-assessment data. Finally, we focused on highlight the potential of computer vision technology as a new approach to screening and assessing patients with advanced knee osteoarthritis without considering early-stage knee osteoarthritis that could impact motion function. In future research, it is crucial to discuss the application of computer vision technology in enabling the physical condition assessment of individuals in the early stages of knee osteoarthritis. If proven feasible, this could offer a new and viable approach to providing self-assessment for all knee osteoarthritis patients.

In summary, our study introduces an innovative idea for self-assessment of the physical condition of advanced KOA patients caused by articular cartilage degeneration in older adults and provides evidence for its viability. Unlike expensive optical motion capture devices for clinical motion analysis, our study enables convenient at-home assessments using smartphones, reducing entry barriers and enhancing accessibility. This approach holds the potential to provide convenient and reliable avenues for self-assessment for knee osteoarthritis patients.

## Data availability statement

The original contributions presented in the study are included in the article/Supplementary Materials, further inquiries can be directed to the corresponding authors. Our method is not specific to the datasets used in this study, and users can train and test the deep-learning model on any relevant video data. We completed the study using Python 3.9. The operating system was Windows 10, and the graphics processing unit (GPU) was an NVIDIA GeForce RTX 3060 graphics card. The source code is available at: https://github.com/ZhengkuanZhao/Self-assessment-of-knee-osteoarthritis-patients/.

## Ethics statement

The studies involving humans were approved by Medical Ethics Committee of Tianjin Hospital, Tianjin, China. Institutional Review Board number: IRB 2023 Medical Ethics Review 066. The studies were conducted in accordance with the local legislation and institutional requirements. The participants provided their written informed consent to participate in this study. Written informed consent was obtained from the individual(s) for the publication of any potentially identifiable images or data included in this article.

## Author contributions

ZZ: Conceptualization, Data curation, Formal analysis, Investigation, Methodology, Resources, Supervision, Validation, Writing – original draft, Writing – review & editing. TY: Conceptualization, Formal analysis, Investigation, Methodology, Resources, Software, Validation, Visualization, Writing – original draft, Writing – review & editing. CQ: Conceptualization, Formal analysis, Investigation, Methodology, Resources, Validation, Visualization, Writing – original draft, Writing – review & editing. MZ: Data curation, Investigation, Methodology, Resources, Software, Validation, Writing – review & editing. FZ: Data curation, Investigation, Resources, Validation, Writing – review & editing. BL: Conceptualization, Funding acquisition, Investigation, Project administration, Supervision, Validation, Writing – original draft, Writing – review & editing. JL: Conceptualization, Funding acquisition, Investigation, Methodology, Project administration, Supervision, Validation, Writing – original draft, Writing – review & editing.
