# Supplementary material for: Exploring the potential of the sit-to-stand test for self-assessment of physical condition in advanced knee osteoarthritis patients using computer vision
Source: Front Public Health. 2024 Feb 7;12:1348236. doi: 10.3389/fpubh.2024.1348236 (PMC10880867; doi:10.3389/fpubh.2024.1348236)
Supplement: Supplementary file 1 [file Table_1.DOCX]

**The “Supplemental information.docx” file:**

It includes raw data results of quantitative analysis and the seventeen key positions obtained from VideoPose. It also presents the network architectures of AlphaPose and VideoPose, as well as the schematic diagram of our overall research process.

| **S1 Table Quantitative analysis of coefficient** | | | | | | | | |
| --- | --- | --- | --- | --- | --- | --- | --- | --- |
|  | Mild stiffness | | Severe stiffness | | Mild limitations of physical function | | Severe limitations of physical function | |
|  | cA | cD | cA | cD | cA | cD | cA | cD |
| right hip | 35340.5 | 141.8 | 34916.4 | 166.7 | 34579.1 | 164.3 | 35401.8 | 142.4 |
| right knee | 36636.9 | 123.8 | 37250.9 | 123.7 | 37289.7 | 120.6 | 36811.2 | 126.7 |
| left hip | 33442.8 | 153.7 | 33394.1 | 153.4 | 34247.9 | 159.7 | 31980.3 | 149.6 |
| left knee | 33863.3 | 132.4 | 34481.2 | 121.8 | 34087.1 | 139.6 | 33780.9 | 117.1 |

Stiffness shows the results of the quantitative analysis of stiffness between mildly stiff patients and severely stiff patients. Physical function shows the results of the quantitative analysis of stiffness between mild limitations of physical function patients and severe limitations of physical function. cA and cD represent the absolute values at each joint position of the approximation coefficients and detail coefficients of Different stiffness levels and different motor functions of patients.

**Get the seventeen key positions**

We obtained seventeen key positions include "interfemoral," "right hip," "right knee,' 'right foot,' 'left hip,' 'left knee,' 'left foot,' 'spine,' 'thorax,' 'nose,' 'head,' 'left shoulder,' 'left elbow,' 'left wrist,' 'right shoulder,' and 'right elbow.'

1. Wang H, King B, Yang R. The Development and Psychometric Evaluation of the Perceived Physical Literacy for Chinese Elderly Questionnaire (PPLCEQ). *Gerontologist* 2022.

**S1 Fig The network architecture of AlphaPose.**


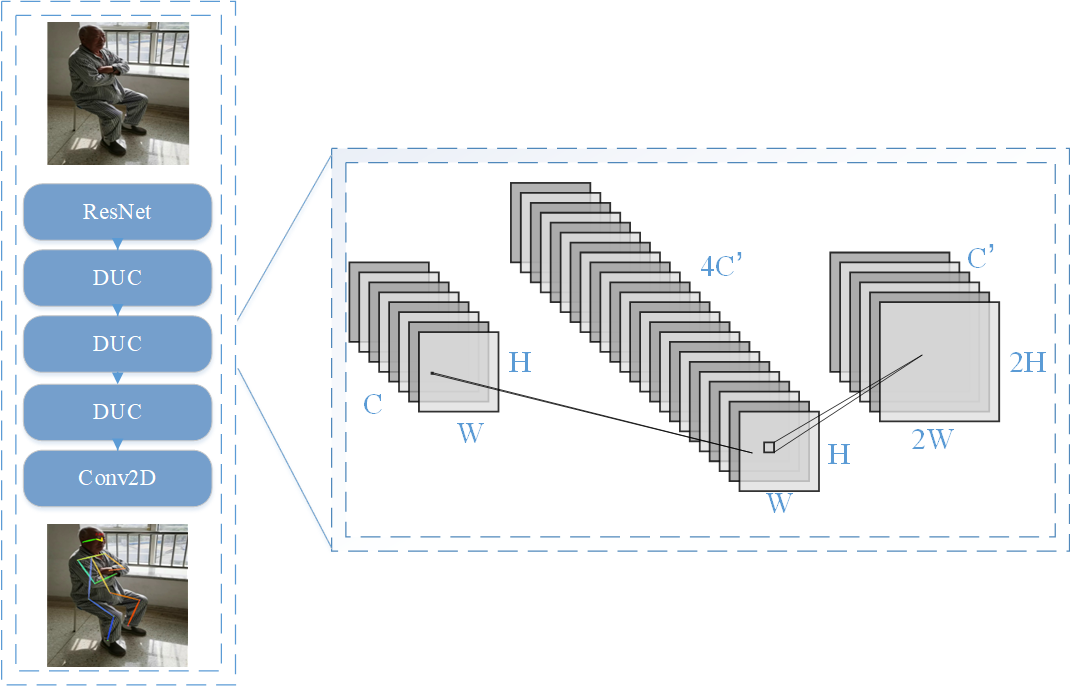


The inputs are physical function test videos. The network architecture begins with adopting ResNet as the backbone, the foundation for feature extraction from the video frames. Then, three Deformable Units of Convolution (DUC) are applied for up-sampling, as depicted on the right side. These modules are stacked one after another, sequentially increasing the resolution of the feature maps. The H and W represent the height and width of the picture and C represents the number of the channel. The DUC modules ensure that the up-sampled feature maps maintain the spatial relationships and capture the intricate details necessary for accurate human pose estimation. Finally, a 1 × 1 convolutional layer generates heatmaps representing the two-dimensional body pose. This convolutional layer processes the high-resolution feature maps obtained from the DUC modules and produces heatmaps that highlight the locations of the body pose. The outputs are physical function test videos with two-dimensional body position markers.

**S2 Fig The network architecture of VideoPose.**


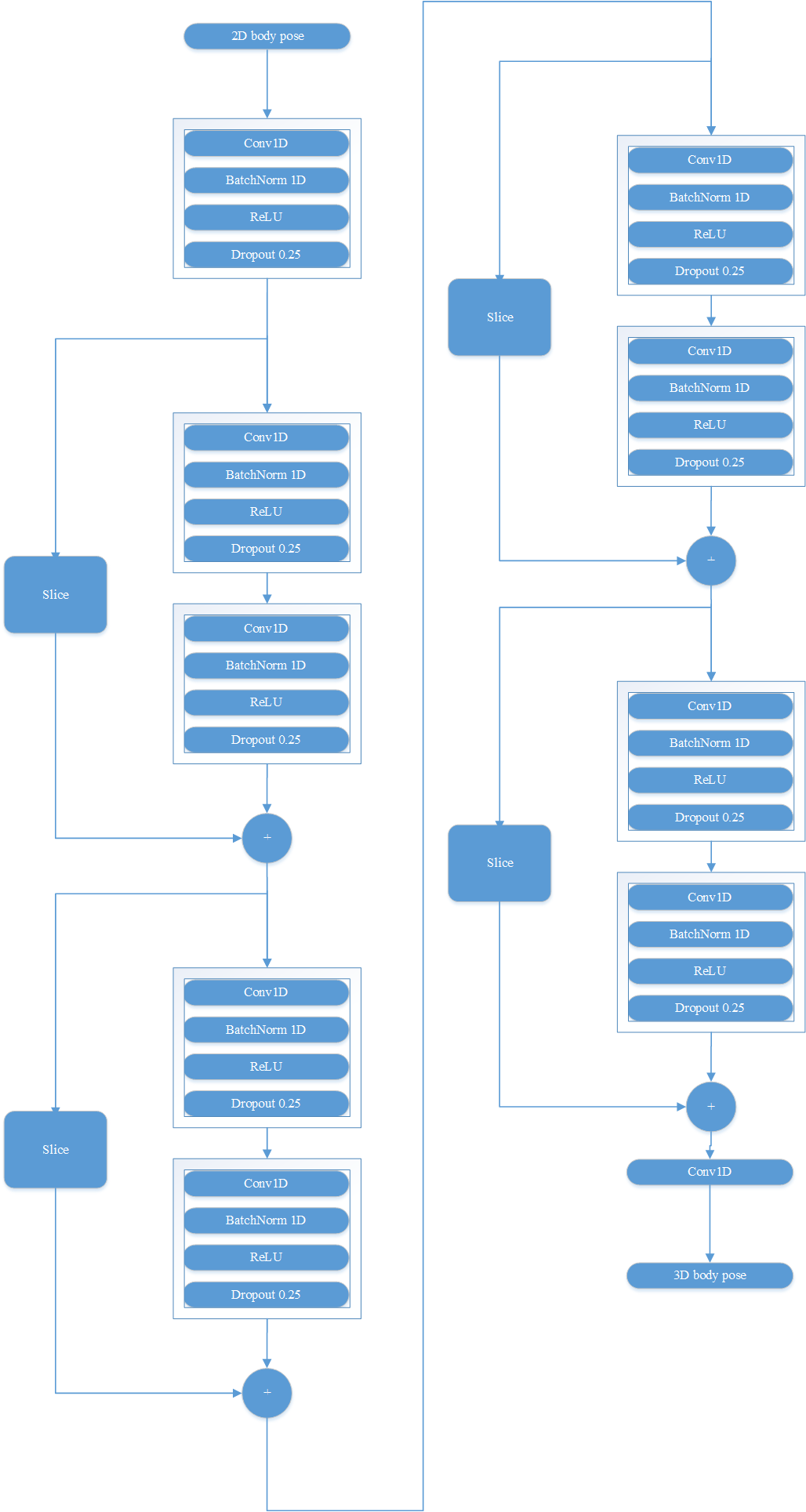


The inputs are two-dimensional body positions, representing the spatial coordinates of the body positions in the videos. We use valid convolutions, which means that the convolutional operations are performed to ensure the output remains within the valid input boundaries. Then we employ residual slicing. Residuals represent the difference between the predicted output and the ground truth values. In our architecture, we symmetrically slice the residuals into left and right parts, ensuring they match the shape of the subsequent tensors. We ultimately generate three-dimensional body positions as outputs by leveraging valid convolutions and applying residual slicing.
