# Supplementary material for: Exploring the potential of the sit-to-stand test for self-assessment of physical condition in advanced knee osteoarthritis patients using computer vision
Source: Front Public Health. 2024 Feb 7;12:1348236. doi: 10.3389/fpubh.2024.1348236 (PMC10880867; doi:10.3389/fpubh.2024.1348236)
Supplement: Supplementary file 2 [file Data_Sheet_1.ZIP › Supplementary Materials/Description of the Supplementary Materials.docx]

**Description of the content in the compressed package “Supplementary Materials”**

# The “Time series data analysis results” folder:

**It contains our study's qualitative and quantitative data analysis results..**

The “STS Left (stiffness).ipynb” file: The data analysis results of "left hip" and "left knee" positions between KOA patients with different stiffness levels.

The “STS Right (stiffness).ipynb” file: The data analysis results of "right hip" and "right knee" positions between KOA patients with different stiffness levels.

The “STS Left (physical function).ipynb” file: The data analysis results of "left hip" and "left knee" positions between KOA patients with different physical function.

The “STS Right (physical function).ipynb” file: The data analysis results of "right hip" and "right knee" positions between KOA patients with different physical function.

Please be aware that .ipynb files must be opened using Python or software that supports Python to ensure accurate interpretation and execution.

# The “Demographic characteristics.xlsx” file:

**It is the demographic characteristics of patients.**

Sex :1: Male ; 2: Female

WOMAC (B) - Joint Stiffness: 0: Mild Stiffness ; 1: Severe Stiffness

WOMAC (C) - Physical Function: 0: Mild Limitation ; 1: Severe Limitation

**The “Time series data” folder：**

It includes three-dimensional spatiotemporal data reflecting the variation of key joint angles over time, obtained from the patients.
